# Nutrition assessment of vitamin A and vitamin D in northeast Chinese population based-on SPE/UPLC/PDA

**DOI:** 10.1186/s40795-018-0219-x

**Published:** 2018-03-27

**Authors:** Maoqing Wang, Hongyu Chen, Shanshan Du, Xinxin Guo, Jiali Zhao, Changhao Sun, Ying Li

**Affiliations:** National Key Disciplines of Nutrition and Food Hygiene, Department of Nutrition and Food Hygiene, School of Public Health, Harbin Medical University, 157 Baojian Road, Nangang District, Harbin 150081 People’s Republic of China

## Abstract

**Background:**

The aims of the current study were to assess the nutritional status of 25OHD3 and retinol in a northern Chinese population using our established reliable method for the simultaneous determination of serum 25OHD3 and retinol.

**Method:**

We established a reliable method for the simultaneous determination of 25OHD3 and retinol using SPE and UPLC/PDA; measured the serum levels of 25OHD3 and retinol in elementary school students, middle school students, and adults (*n* = 1181) in northern China; and assessed their nutritional status.

**Results:**

Our method had good precision, detection limit, and linear quantitative range and could process 100 samples within 12 h. The average levels of 25OHD3 and retinol were 16.1 ± 6.7 ng/ml and 328.1 ± 117.1 ng/ml, respectively, in all samples. VD deficiency was common, with a prevalence > 60% in all three age groups, and the high prevalence of VA deficiency (26.1%) was observed only in the elementary school students.

**Conclusions:**

Vitamin A supplementation should be considered for elementary school students, and vitamin D supplementation is highly recommended for all age groups in Harbin. Our method could be widely adopted in population-based studies and clinical practice.

**Electronic supplementary material:**

The online version of this article (10.1186/s40795-018-0219-x) contains supplementary material, which is available to authorized users.

## Background

Deficiencies in Vitamin A (VA) and vitamin D (VD) are important public health problems and the incidences of these deficiencies remain very high throughout the world [1, 2]. Women of childbearing age and children are particularly at risk in both developing and developed countries [3]. Rapid and accurate assessments in VA and VD deficiencies were urgently needed for their detrimental effects on human health. Megavitamin therapy and indiscriminate use of vitamins may induce hypervitaminosis or toxicity from vitamins A [4] and D [5], especially dangerous during pregnancysince [6] since vitamins A and D are the fat-soluble vitamins, which are readily stored in the body. Therefore, assessments of VA and VD status at both the individual and population level are important for diagnostic and monitoring supplementation purposes [7]. For this reason, there is a need for development of reliable biomarkers for assessment of vitamin A and D nutritional status.

Measurement of the liver VA concentration is considered the best indicator of VA nutritional status, but it is not feasible in human studies [8]. In normal healthy human serum, retinol is homeostatically controlled and its concentration will not drop until body stores are significantly compromised, thus it tends to be the most used indicator in human studies [9, 10]. It was well know that 25OHD_3_ and 25OHD_2_ are the two main metabolites of VD. More and more evidences suggested a stronger biological effect of 25OHD_3_ than 25OHD_2_ in humans [11]. Furthermore, except in the case of ingestion of vitamin D_2_ drug preparations, serum 25OHD_2_ is not observed. Circulating active VD (1,25-dihydroxyvitamin D_3_) has a short half-life and is closely linked to parathyroid hormone production. Serum levels of 1,25-dihydroxyvitamin D_3_ do not reflect VD status. Therefore, serum 25OHD_3_ is the best and the most widely used indicator of nutritional VD status.

High-performance liquid chromatography (HPLC) and HPLC/ MS (mass spectrometer) have been used for simultaneous determination of serum 25OHD_3_ and retinol in serum [12–14]. Current methods, however, exhibit some disadvantages and/or limitations [7]. For instance, VA and 25OHD_3_ are extracted from serum by liquid–liquid extraction (LLE) [10]. Compared with solid-phase extraction (SPE), the pretreatment of serum samples by LLE requires a number of manual transfers of the sample between vials and steps, which are prone to error. Moreover, LLE is laborious, time-consuming and requires appreciable organic solvent. Therefore, SPE is now widely accepted as an attractive alternative to LLE and where there constituted sample residue is separated and analyzed by HPLC. Compared with UPLC, HPLC does require more solvent and separation takes longer [15]. As with LC, LC-MS is an accurate testing method for 25OHD_3_ and retinol. However, compared with LC, the cost of analysis based on LC/MS is more expensive. A rapid, accurate, and low-cost detection method for serum retinol and 25OHD_3_ is needed for both population studies and clinical practice.

Considering all above conditions, we developed a simple and rapid method for simultaneous determination of serum 25OHD_3_ and retinol using SPE, UPLC and diode array detection (PDA). Then, this method was used for the assessment of VA and VD deficiency in three age groups (aged 6–8, 11–13 and 18–65) in Harbin, China.

## Methods

### Establishment of a rapid detection method of vitamin a and vitamin D based-on SPE/UPLC/PDA

#### Reagents and chemicals

Acetonitrile and methanol (chromatographic grade) were purchased from Honeywell Burdick & Jackson (Muskegon, MI, USA). Formic acid and ethyl acetate (analytical grade) were from the Beijing Reagent Company (Beijing, China). The ultrapure water was prepared by an ultra clear system (PURELAB Ultra, Veolia Water Solutions & Technologies, France). Retinol and 25OHD_3_ were supplied by Sigma-Aldrich (Sigma-Aldrich Co Ltd., Shanghai, China).

#### Preparation of standard solutions

Stock solutions of retinol (1 mg/ml) and 25OHD_3_ (1 mg/ml) were prepared with ethanol and stored in amber glass vials at − 20 °C until analysis. Working solutions in acetonitrile for both standards were prepared in volumetric flasks (10 ml) in the concentration ranges, 2–100 ng/ml and 20–750 ng/ml, for 25OHD_3_ and retinol, respectively. The calibration curves were based on 5 concentration levels for each standard compound.

#### Sample preparation

First, 200 μl acetonitrile was added to each 100 μl serum sample. After shaking for 2 min, the samplewas centrifuged at 12000 rpm for 10 min at 4 °C. The supernatant and 100 μl water were both transferred to a new tube. After vortexing for 60s and centrifuging at 12000 rpm for 10 min at 4 °C, the mixed solution was transferred to SPE column (Waters OASIS HLB μ Elution plates, Waters), which had been previously activated with 200 μl ethyl acetate, 200 μl methanol, and 200 μl water. The SPE μ Elution plate was then washed with 100 μl water and 100 μl methanol (50% *v*/v). The fractions containing 25OHD_3_ and retinol were eluted from the column using 100 μl acetonitrile and collected in a 96 well sample plate. All the extraction procedures were performed under yellow light to minimize decomposition of the analytes.

#### UPLC/PDA conditions

Samples were analyzed using a Waters ACQUITY UPLC system equipped with an ACQUITY photodiode array (PDA) (Waters Corporation, Milford, MA, USA). Separation of the extracted sample (2 μL) was carried out with a Waters ACQUITY UPLC BEH C18 column (2.1 × 100 mm; 1.7 μm particle size) and a PDA detector. The temperature of UPLC column was maintained at 45 °C. The mobile phase was a mixture of 85% acetonitrile (eluent A) and 15% water containing 0.1% formic acid (eluent B) at a flow rate of 0.35 mL/min. The PDA detector covered the wavelength range 220–360 nm to permit simultaneous detection of retinol and 25OHD_3_ at 325 nm (retinol) and 256 nm (25OHD_3_). The sensitivity, signal linearity, precision, recovery and limits of detection and quantitation were calculated. The lower limits of detection (LLOD) and quantitation (LLOQ) were determined by serial dilutions of pooled serum containing 25OHD_3_ and retinol.

#### Intra- and inter-assay precision and accuracy

The concentrations for the quality control (QC) samples were selected to encompass the entire range of the calibration curves corresponding to the levels anticipated to occur in most samples: low (L), medium (M) and high (H). Replicate analyses (*n* = 6) of three QC samples were conducted to assess the intra-assay precision and accuracy. The precision was evaluated as the coefficient of variation (CV %) and the accuracy was calculated as the bias or percent deviation between the nominal and measured concentrations. The recovery of adding standard was assessed by comparing the concentrations of 25OHD_3_ and retinol in serum samples before and after the addition of known amounts of 25OHD_3_ and retinol. Five serum samples with 25OHD_3_ and retinol concentrations ranging from 10 to 50 and 50–400 ng/mL were used. The concentrations of 25OHD_3_ and retinol added to each sample were 10, 20 and 50 ng/mL, and 50, 100 and 400 ng/mL, respectively.

The linearity measuring ranges were evaluated by constructing standard curves for 25OHD_3_ and retinol. The response was considered to be linear if the correlation coefficient was greater than 0.99.

#### Verification of UPLC/PDA on a UPLS/Xevo™ TQ MSMS

Our method using SPE/UPLC/PDA requires verification through analysis on another platform or the use of external QA samples. LC-MS/MS has already been introduced as a simple, rapid, and robust reference method for the determination of steroid hormones, such as 25OHD_3_. Therefore, LC-MS/MS was used as the reference method to validate our established method. The 30 participants were randomly selected, pretreatment and separated the same as above method. Mobile phase A included 15% water + 2% formic acid, and mobile phase B included 85% acetonitrile + 2% formic acid. But, the serum concentrations of 25OHD3 and retinol were measured on a UPLS/Xevo™ TQ MSMS (Waters Corporation, Milford, MA, USA) in ESI positive mode. The conditions of the Xevo™ TQ MSMS are shown in Additional file 1: Table S1. To illustrate the magnitude of differences between UPLDA/PDA and UPLC/TQ MSMS, the differences, expressed as a percentage of the LC-MS/MS value, were plotted [16]. Wilcoxon matched-pairs signed-rank test was used to analyze the differences between the two methods.

### Nutrition assessment of VA and VD in northeast Chinese population

#### Study subjects

One across-sectional study was carried out from June to July 2013 in Xiang fang district, Harbin. It is the biggest city in northern China (44 °N~ 46°N). Using the group sampling method, a total of 600 adults (aged 35–65) from Haping health service center, 274 students (aged 12–17) from Rongzhi middle school, and 307 students (aged 6–11) from Xinghua elementary school were randomly selected. A total of 1181 participants, 600 adults, 274 middle school students and 307 elementary school students were included in current students. Fasting blood samples were collected from all participants before 9:00 am after ≥10 h fasting. All blood samples were centrifuged at 2500×g for 15 min at room temperature within 30 min after collection, then the supernatant was stored at − 80 °C until measurements. Serums were pre-treated and 25OHD_3_ and retinol were determined by the described above method. All experiments including all relevant details were performed in accordance with relevant guidelines and regulations.

#### Nutrition assessment

Serum retinol and 25OHD_3_ were used in the assessment of VA and VD nutrition status. Sufficient, insufficient, mild deficient and deficient VA were defined as serum retinol ≥300, 200~ 300, 100~ 200, and < 100 ng/ml. Sufficiency, insufficiency, deficiency, and severe deficiency in VD were defined as serum 25OHD_3_ ≥ 30, 20~ 30, 10~ 20, and < 10 ng/ml.

## Results

### Quantitative detection of 25OHD3 and retinol based on SPE/UPLC/PDA

**Fig. 1:**
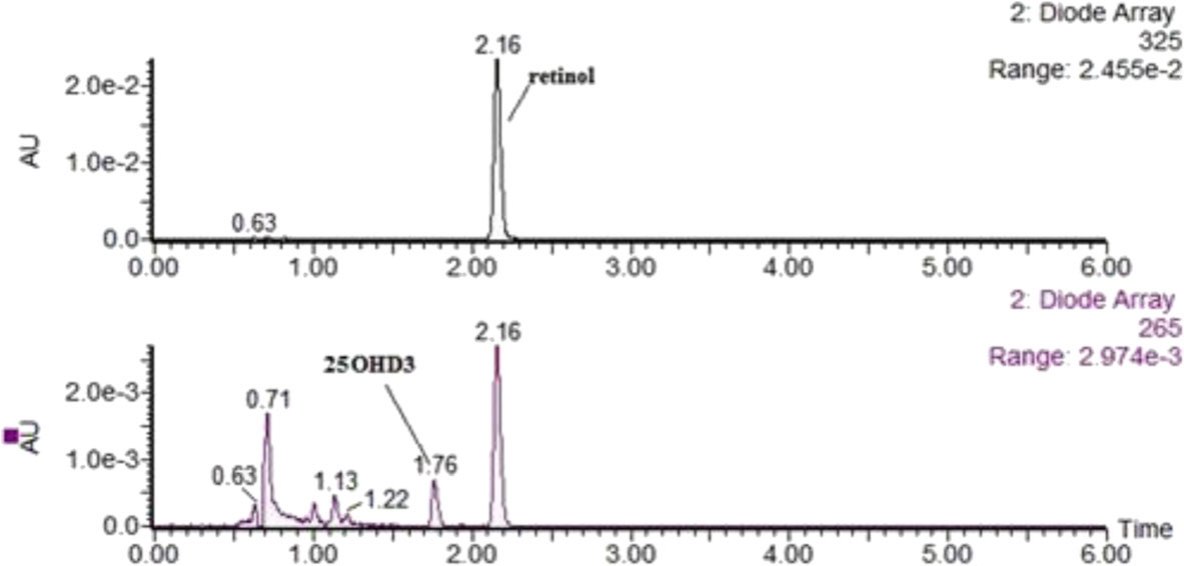
Chromatograms of retinol and 25OHD_3_

As shown Fig. 1, 25OHD_3_ and retinol were separated by UPLC C18 column in 6 min. The retention times of 25OHD_3_ and retinol were 1.76 and 2.16 min (Fig. 1). Acetonitrile (200 μl) was added to serum (100 μl) for deproteinization. Therefore, the polarity of the supernatant was low (acetonitrile: water ≥2:1). When sample solutions were directly transferred to the SPE column, 25OHD_3_ and retinol may not be completely retained on the column. To decrease the potential losses of 25OHD_3_ and retinol in sample processing, 100 μl water was added to the acetonitrile supernatant to decrease the polarity of the solution. Based on the same reasoning, 50% methanol was used as a second eluant. The average recoveries for 10, 20 and 50 ng/ml 25OHD_3_ were 97.8%, 101.7% and 94.4%, respectively. The average recoveries for 50, 100 and 400 ng/ml retinol were 98.1%, 97.2% and 96.5%, respectively. Therefore, both compounds exhibited acceptable recoveries for the Oasis HLB SPE 96-well elution plate.

**Fig. 2:**
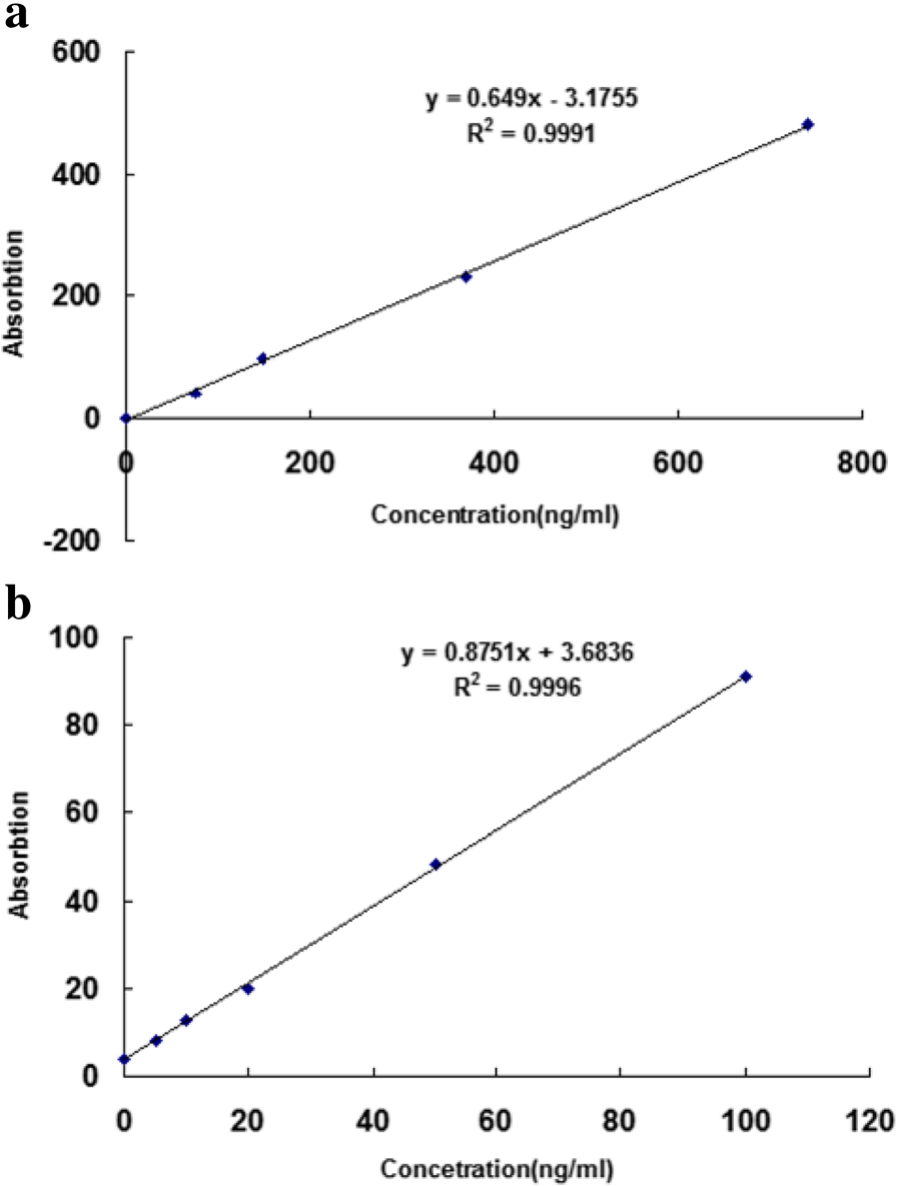
Calibration graphs for retinol and 25OHD_3_. **a**: retinol; **b**: 25OHD_3_

The relative standard deviation (*n* = 6) of the inter-day precision for 25OHD3 and retinol were 4.69% and 3.72%. The relative standard deviation of intra-day precision for 25OHD3 and retinol were 5.26% and 4.62%. Also good linearity of quantification (R2 > 0.99) were obtained over the concentration ranges 2 to100 ng/ml (25OHD_3_) and 25 to740 ng/ml (retinol). The regression equations were y = 0.877× + 3.6836 (Fig. 2a) and y = 0.649× − 3.1755 (Fig. 2b). The LODs for 25OHD_3_ and retinol were 1 ng/ml and 25 ng/ml, respectively.

### Quantitative detection of 25OHD3 and retinol by SPE/UPLC/XEVO TQ MS

The relative standard deviation (*n* = 6) and the inter-day precision for 25OHD_3_ and retinol were 3.63% and 2.15%, respectively. The relative standard deviation of the intra-day precision for 25OHD3 and retinol were 4.65% and 2.99%, respectively. Good linearity for the quantification (R2 > 0.99) was obtained over 2.5 to 100 ng/ml (25OHD_3_) and 4 to 800 ng/ml (VA). The regression equations were y = 14.3959× + 651.83 (VA Additional file 1: Figure S1A) and y = 15.77× − 26.52 (25OHD_3_ Additional file 1: Figure S1B). The LODs for 25OHD_3_ and retinol were 1 ng/ml and 4 ng/ml, respectively.

### Comparison of UPLS/PDA and UPLC/MS

Plots of the % differences against the LC-MS/MS values were applied to 30 samples (Additional file 1: Figure S2). No significant differences (*p* > 0.9) in 25OHD3 and retinol were observed between UPLC/PDA and UPLC/MS by Wilcoxon matched-pairs signed-rank test. These results indicate that our method was reliable in simultaneously determining serum 25OHD3 and retinol.

### Nutritional assessment of VA and VD in a northern Chinese population

Serum 25OHD_3_ and retinol were determined in 307 elementary school students, 274 middle school students, and 600 adults. The mean levels of serum 25OHD_3_ were 16.1 ± 6.7 ng/mL, 17.8 ± 7.3 ng/mL, 15.3 ± 5.7 ng/mL, and 15.4 ± 6.1 ng/mL in the entire cohort, elementary school students, middle school students, and adults, respectively (Table 1). Significant differences were observed between these three groups (*P* < 0.001). The means between any two groups also differed (all *P*-values = 0.000), except between adults and middle school students (*P*-values = 1.000). These results indicate that elementary school students have higher serum levels of 25OHD_3_ compared with middle school students and adults. As shown in Table 2, with regard to serum vitamin D, severe deficiency (< 10 ng/mL) and deficiency [(10–20) ng/mL] were detected in 12.7% and 64.7% of participants, respectively, and 19.4% and 3.2% of participants were vitamin D-insufficient [(20–30) ng/mL] and -sufficient (> 30 ng/mL). Although elementary school students had the highest serum 25OHD_3_ among the three groups, over 65% (8.5% severe deficiency and 58.3% deficiency) were considered deficient. Adults and middle school students were even more lacking in vitamin D.

**Table 1:** Serum 25OHD3 and retinol levels of all participants

Participants	25OHD_3_ (ng/ml)	*P*-value	Retinol (ng/ml)	*P*-value
Elementary school students	17.8 ± 7.3	0.000 ^a^	255.5 ± 78.2	0.000 ^a^
Middle school students	15.4 ± 6.1	0.000 ^b^	319.8 ± 113.3	0.000 ^b^
Adults	15.3 ± 5.7	0.000 ^c^	369.1 ± 116.8	0.000 ^c^
Total	16.1 ± 6.7	1.000 ^d^	328.1 ± 117.1	0.000 ^d^

^a^ results of One-way ANOVA for the comparison of elementary school students, middle school students, and adults

^b^ results of One-way ANOVA (Bonferroni correction) for the comparison with elementary school students

^c^ results of One-way ANOVA (Bonferroni correction) for the comparison with elementary school students

^d^ results of One-way ANOVA (Bonferroni correction) for the comparison with middle school students

**Table 2:** Nutrition assessment of Vitamin A in three age groups

Participants	Deficiency	Marginal deficiency	Insufficiency	Sufficiency	*P*-value
Elementary school students	3(1.0)	77(25.1)	143(46.6)	84(27.4)	0.000^a^
Middle school students	0(0)	33(12.0)	103(37.6)	138(50.4)	0.000^b^
Adults	0(0)	22(3.7)	156(26.0)	422(70.3)	0.000^c^
Total	3(0.3)	132(11.2)	402(34.0)	644(54.5)	0.000^d^

Note: all data were shown in n (%); deficiency: < 100 ng/ml; marginal deficiency: 100~ 200 ng/ml; insufficient: 200~ 300 ng/ml; sufficient: ≥ 300 ng/ml

^a^ results of Chi-Square tests for the comparison of elementary school students, middle school students, and adults (the cut-off value of *P* is 0.05)

^b^ results of Chi-Square tests for the comparison with elementary school students (the cut-off value of *P* with Bonferroni correction is 0.05/3)

^c^ results of Chi-Square tests for the comparison with elementary school students (the cut-off value of *P* with Bonferroni correction is 0.05/3)

^d^ results of Chi-Square tests for the comparison with middle school students (the cut-off value of *P* with Bonferroni correction is 0.05/3)

The mean levels of serum retinol were 328.1 ± 117.1 ng/mL, 255.5 ± 78.2, 319.8 ± 113.3, and 369.1 ± 116.8 ng/mL in the entire pool, elementary school students, middle school students, and adults, respectively. Based on the multiple comparisons, statistically significant differences in the means of serum retinol concentration between any two groups (all *P*-values = 0.000) were observed. Unlike serum 25OHD_3_, the highest and lowest levels of serum retinol were seen in adults and elementary school students, respectively. As shown in Tables 3, 0.3% and 11.2% of participants had levels under 100 ng/mL (deficiency) and 100~ 200 ng/mL (marginal deficiency), respectively. Also, 34.0% and 54.5% of participants had insufficient (200–300) ng/mL and sufficient (> 300 ng/mL) serum VA, respectively. More than 88% of middle school students and adults had sufficient nutritional status. However, 1.0% and 25.1% of elementary school students were considered to have deficiency and mild deficiency, respectively. The VA nutritional status of the population in Harbin was better than that of VD, and most of the population had good nutritional sufficiency.

**Table 3:** Nutrition assessment of Vitamin D in three age groups

Participants	Severe deficiency	Deficiency	Insufficiency	Sufficiency	*P*-value
Elementary school students	26(8.5)	179(58.3)	84(27.4)	18(5.9)	0.000^a^
Middle school students	47(17.2)	168(61.3)	53(19.3)	6(2.2)	0.001^b^
Adults	76(12.7)	416(69.3)	94(15.7)	14(2.3)	0.000^c^
Total	150(12.7)	764(64.7)	229(19.4)	38(3.2)	0.110^d^

Note: all data were shown in n (%); severe deficiency: < 10 ng/ml; deficiency: 10~ 20 ng/ml; insufficient: 20~ 30 ng/ml; sufficient: ≥ 30 ng/ml

^a^ results of Chi-Square tests for the comparison of elementary school students, middle school students, and adults (the cut-off value of *P* is 0.05)

^b^ results of Chi-Square tests for the comparison with elementary school students (the cut-off value of *P* with Bonferroni correction is 0.05/3)

^c^ results of Chi-Square tests for the comparison with elementary school students (the cut-off value of *P* with Bonferroni correction is 0.05/3)

^d^ results of Chi-Square tests for the comparison with middle school students (the cut-off value of *P* with Bonferroni correction is 0.05/3)

## Discussion

VA and VD are essential nutrients, and insufficiency of these vitamins is associated with several acute and chronic diseases. Yet, the incidence of VA and VD deficiency has remained very high throughout the world. Among all causes of disease, it is the most frequently overlooked. Therefore, a high-throughput, low-cost method for determining retinol and 25OHD_3_ is needed. The key contribution of our study was the establishment of an easy and fast method for routine measurements of 25OHD_3_ and retinol, based on SPE and UPLC/PDA, with which we assessed the nutritional status of VA and VD in a northern Chinese population.

Based on optimized instrument parameters, our method showed good precision and excellent accuracy in simultaneously determining the metabolites of VA and VD. The average levels of retinol and 25OHD_3_ in serum range from 100 to 500 ng/ml and 10–35 ng/ml, respectively; thus, the sensitivity (retinol: 20 ng/ml; 25OHD_3_: 2 ng/ml) of this new method is satisfactory for population-based and clinical detection. Compared with LC/MC, this UPLC/PDA method is less expensive for large population studies. It also decreased the use of toxic organic solvents and shortened the sample preparation time. Samples could be prepared within 15 min, and the separation time was within 3 min. The proposed method, based on SPE/UPLC/PDA, can process 100 serum samples within 12 h. If increased capacity is required, the system can run reliably overnight, unsupervised and doubling sample throughput.

Another important advantage of this method is its lower sample volume consumption (100 μl per sample), which is an important factor analytically and clinically. Compared with liquid-liquid extraction, the cost of SPE cartridges can be offset by the savings in labor and reagent expenses. Therefore, this simple and fast method for measuring serum retinol and 25OHD3 is suitable and can be recommended for nutritional assessments in population-based studies and clinical practice.

VA and VD are necessary to maintain health, and their deficiency may cause various conditions [17]. Describing the status of VA and VD in a population has tremendous significance for public health. Serum retinol and 25OHD_3_ were determined in 307 elementary school students, 274 middle school students and 600 adults in this study. According to WHO standards, serum retinol concentrations are classified as ≥1.05 μmol/L (300 ng/ml) for normal, 0.70–1.05 μmol/L (200–300 ng/ml) for marginal, and < 0.70 μmol/l (200 ng/ml) for deficient. Elementary school students (aged 6–11 years) had the lowest levels of serum VA (255.5 ± 78.2 ng/ml), the highest prevalence of VA deficiency (1% deficiency; 25.1% marginal deficiency). In contrast, 12% of middle school students and 3.7% of adults were considered marginally deficient. The China National Nutrition and Health Survey (2010–2013) has estimated the prevalence of serum VA deficiency (< 200 ng/mL) to be 8.04% in 6–11-year-olds [18]. The mean levels VA in the population of Harbin City (aged 6–11 years: 255.5 ng/ml; aged 11–17 years: 319.8 ng/ml) were lower than the national mean levels (aged 6–11 years: 1.46 μmol/L (417 ng/ml); 12–17 years: 1.54 umol/L (440.4 ng/ml)). The prevalence of mild VA deficiency (25%) in the Harbin population (ages 6–11 years) was higher than the national average (16.54%). But, the prevalence of vitamin A deficiency (1% and 0%) in Harbin (ages 6–11 years and 12–17 years, respectively) was lower than the national average (8.04% and 7.18%) [19]. Previous studies have indicated that VA deficiency decreases with age [20]. Our results indicated a similar trend, wherein severe VA deficiency was only observed in younger school-age students (6–11 years), and marginal deficiency was observed in elder students (12–17 years), but there was no deficiency in adults.

VA plays an important role in the adverse effects of virus and bacterial infections [21] and as parasitic infections [22]. Vitamin A deficiency can affect immune function and lead to higher morbidity and mortality due to respiratory and digestive tract infections in children [23, 24]. Therefore, we strongly recommended appropriate VA supplementation for elementary school students in Harbin.

Previous studies have documented severe VD deficiency in all age groups worldwide. In China, the prevalence of serum 25OHD_3_ deficiency is higher than 50% in all age groups [25, 26], reaching 85.8%, 97%, and 89.7% in school-aged students, adolescents, and adults, respectively, in some remote and low-income regions [27–29]. The prevalence of vitamin D deficiency in schoolchildren (ages 7 to 11 y) is 56.4% in 2013 [30] and 45.85% of (ages 1 to 10 y) [31] in Harbin. The prevalence of suboptimal vitamin D (vitamin D deficiency and insufficiency) is 74.7% (ages 20 to 74 y) [32] and 87.35% (ages 1 to 10 y) [31] in Harbin. In our study, we observed a high prevalence of VD deficiency (66.8%, 78.5%, and 82.0%, respectively) was observed in elementary school students, middle school students, and adults in this study. The major natural source of vitamin D is photosynthesis in the skin following ultraviolet B solar irradiation. Harbin is located at high latitudes and in a cold region (N44–46°). The lower level of 25OHD_3_ from skin photosynthesis in Harbin residents can be explained in part by the shortage of sun exposure and less outdoor activity during long winters.

A small amount of VD is acquired from food. The most significant dietary sources of VD are oily fish and cod liver oil. However, the typical Harbin diet lacks these foods. Low dietary vitamin D intake, combined with the lack of skin synthesis for half of the year, is reflected in the disturbingly high prevalence of VD insufficiency throughout Harbin. Vitamin D deficiency is associated with skeletal disease, rickets [33], and osteomalacia. Several observational studies have shown that VD deficiency also increases the probability of chronic non-communicable diseases, such as stroke [34], diabetes [32], and hypertension [35, 36]. Therefore, monitoring and assessing the status of VA and VD in the population has great public health significance. Based our results, we strong recommended VD supplementation for all three age groups in Harbin.

## Conclusions

Our method is a simple and rapid approach for measuring serum retinol and 25OHD_3_. It is reliable and can be applied in population-based studies and clinical practice. In northern China, VA deficiency is prevalent in elementary school students, and VD deficiency is common in the entire population and is a global public health problem for all age groups. Supplementing vitamin A in elementary school students and VD in all age groups is imperative for improving public health. We strongly recommend this fast and simple method for measuring VA and VD in other populations.

## Additional file

Additional file 1: **Table S1.** TQ MS condition of detecting of serum retinol and 25OHD3. **Figure S1.** Calibration graphs for retinol and 25OHD3. **Figure S2.** Plots of the percentage difference in 25OHD3 and retinol concentrations measured in ULPC/PDA by UPLC/MS-MS. (DOCX 125 kb)

## Abbreviations

LLE: Liquid–liquid extraction
MS: Mass spectrometer
PDA: Photodiode array
QC: Quality control
SPE: Solid-phase extraction
UPLC: Ultra-performance liquid chromatography
VA: Vitamin A
VD: Vitamin D
