# Supplementary material for: Nutrition assessment of vitamin A and vitamin D in northeast Chinese population based-on SPE/UPLC/PDA
Source: BMC Nutr. 2018 Mar 27;4:12. doi: 10.1186/s40795-018-0219-x (PMC7050760; doi:10.1186/s40795-018-0219-x)
Supplement: Supplementary file 1 — Table S1. TQ MS condition of detecting of serum retinol and 25OHD3. Figure S1. Calibration graphs for retinol and 25OHD3. Figure S2. Plots of the percentage difference in 25OHD3 and retinol concentrations measured in ULPC/PDA by UPLC/MS-MS. (DOCX 125 kb) [file 40795_2018_219_MOESM1_ESM.docx]

**Additional file 1: Table S1 TQ MS condition of detecting of serum retinol and 25OHD3**

| **Compounds** | **M/Z(mother ion-**  **daughter ion)** | **Cone voltage(V)** | **Collision energy(eV)** |
| --- | --- | --- | --- |
| **retinol** | 286.27>255.23 | 18 | 10 |
| **25OHD_3_** | 401.10>383.32 | 22 | 10 |
|  | 401.10>90.96 | 22 | 62 |

–1
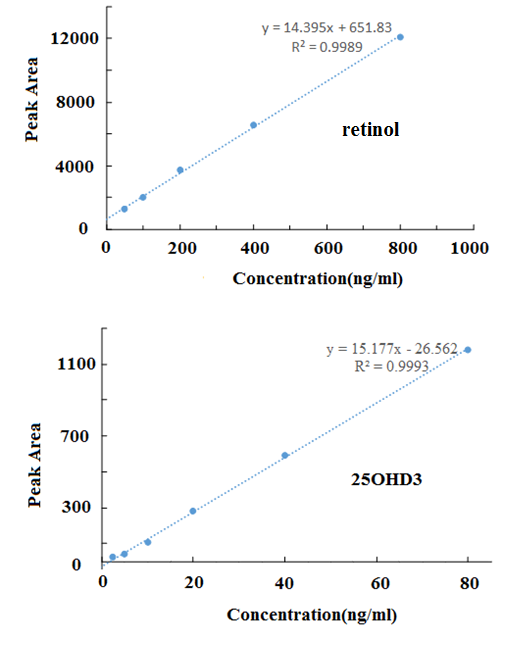


**Additional file 1: Figure S1 Calibration graphs for retinol and 25OHD_3_**


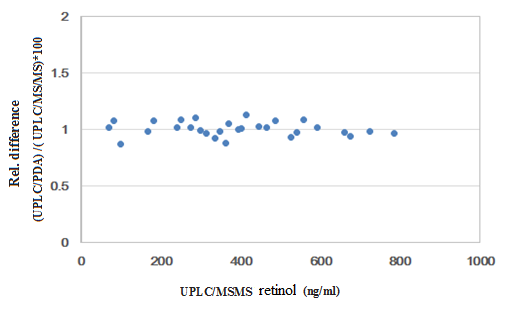


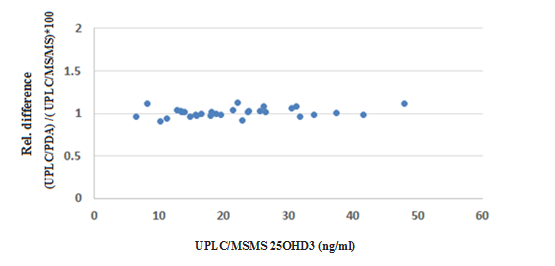


**Additional file 1: Figure S2 Plots of the percentage difference in retinol and 25OHD3 concentrations measured in ULPC/PDA by UPLC/MS-MS.**

–
–
–
